# Editorial: Case reports in aging psychiatry

**DOI:** 10.3389/fpsyt.2023.1305521

**Published:** 2023-11-01

**Authors:** Vincenza Frisardi

**Affiliations:** Geriatric Acute Care, Orthogeriatric Unit and Center for Diagnosis of Cognitive Disorders and Dementia, Istituto di RicerCa a Carattere Scientifico (IRCCS), Azienda Ospedaliera Universitaria Bologna (AOUBO), Bologna, Italy

**Keywords:** aging, psychiatric, neurodegenerative disease, dementia, ageism, mood disorders, depression, late-onset

According to the World Health Organization (WHO)'s projected demographic trends, it is expected that by 2050 about 20% of the population will be over 65 years of age, and 15% of this age group will have psychiatric disorders (1). Neuropsychiatric symptoms, such as depression, anxiety, agitation, and dysphoria, are among the most common psychiatric disorders in old age. Improvements in patient outcomes and the ability to hypothesize alternative diagnoses can result from advances in clinical practice that have been conducted as a result of significant information and new ideas being presented in case reports. The importance of having access to case reports in the emerging, demanding, and challenging field of aging psychiatry has increased. Sharing such valuable and trustworthy information helps us to understand pathogenesis, target new molecular pathways, and improve the quality of care and treatment. Another important aspect of publishing case reports is to produce knowledge that will help the medical community understand more about individualized medicine.

In recent decades, the field of geriatric psychiatry has developed into an independent discipline, incorporating elements of psychiatry, neurology, and geriatric medicine. In this perspective shift, a life-span orientation could be helpful for the recognition of all processes relevant to late-life disorders that often begin in midlife and could be most therapeutically tractable in their early phases. Therefore, the term geriatric psychiatry could be obsolete soon (2). This Research Topic aims to highlight unique cases of patients presenting with an unexpected/unusual diagnosis, treatment outcome, or clinical course. Case reports in this Research Topic provide insight into the differential diagnosis, decision-making, and clinical management of unusual cases and are a valuable educational tool for practitioners and investigators.

## Mood disorders, psychosis, and dementia

In light of a paradigm shift, mood disorders and psychosis in older adults are considered a heterogenous later-life neuropsychiatric syndrome caused by pathophysiologically distinct brain disorders such as Alzheimer's disease (AD), vascular dementia, mixed-dementia, Lewy body dementia (LBD), or frontotemporal dementia, with differences in neuropathology and initial clinical presentation (3). Early diagnosis of LBD can be challenging, particularly in the context of differentiation with the spectrum of mood disorders. Valença et al. show a case of LBD diagnosed as a refractory depression rather than dementia, highlighting how this missed diagnosis could have serious negative consequences (including legal repercussions).

Clinical cases are also crucial to increase knowledge of clinical presentations of psychiatric symptoms in the context of the neurodegenerative process, contributing to a more agile decision-making process. Taomoto et al. report two cases of LBD presenting with delusional infestation and responding better to AChEI than antipsychotics. When delusional infestation (or Ekbom syndrome) occurs, patients are generally diagnosed with delusional disorders, schizophrenia, depression, and dementia and mistaken treatments prescribed.

## Aging and mental health: new frontiers

Late-life neuropsychiatric disorders result from a complex interaction between psychopathology and aging processes affecting brain structure and function (4). To study these interactions, researchers of geriatric mental health have embraced an interdisciplinary approach spanning genetics, molecular biology, cellular physiology, and immunology in addition to maintaining traditional collaborations with geriatric medicine and neurology. Furthermore, new attention has been given to early markers of the preclinical stage of dementia and new underlying biological mechanisms. Neurofascin 186 autoantibodies are known to occur in peripheral nervous system disorders. Recently, additional central nervous system involvement has been reported in conjunction with neurofascin 186 autoantibodies. Hansen et al. (a) suggest a relationship between the neurofascin 186 antibody-associated autoimmunity and the amnestic mild cognitive impairment that occurs in multiple domains, but the lack of immunotherapy interventions in the presented cases requires further studies to confirm and clarify its role. Immune system and blood–brain barrier dysfunction are implicated in the development of dementia syndrome, but their causal role remains not completely understood. Based on some extensive *in silico, in vitro*, and *in vivo* studies, a new mechanistic model of AD as a brain-centric autoimmune disorder has been proposed in response to various initiating stimuli, leading to an interdependent immune- and proteinopathic process (5). Autoimmune-based psychiatric syndromes have emerged as a novel category of disorders. Lessons from autoimmune encephalitis and psychosis have revealed specific immunotherapeutic agents termed first- and second-line immunotherapies also applied in patients presenting autoantibody-associated psychiatric syndromes. Furthermore, randomized, placebo-controlled trials are needed to evaluate immunotherapies in patients with autoimmune-based psychiatric syndromes and to prove their efficacy, inferiority, or superiority over other immunotherapies.

Cancer and neuropsychiatric disorders could share some intriguing etiological commonalities (6). Hansen et al. (b) report an interesting case of dementia syndrome associated with anti-carbonic anhydrase-related protein VIII (CARPVIII) autoantibodies. CARPVIII is reported to be associated with colorectal and breast cancer as well as to paraneoplastic cerebellar degeneration. In addition to dementia's possible autoimmune origin, these authors found circumstantial evidence of a vascular component. Due to anatomical fiber projections, it is conceivable that cognition may be chronically impaired in patients with persistent CARPVIII autoantibodies due to dysfunctional connectivity between the cerebellum, cortex, and hippocampus. Another possibility is that preexisting vascular dementia characterized by severe cerebral microangiopathy is exacerbated by an inflammatory process detected by CARPVIII antibodies [Hansen et al. (b)].

Finally, because of the symptoms overlapping between late-life depressive syndromes and Alzheimer's like dementia, some common underlying mechanisms have been proposed, such as glutamatergic pathway signaling (4). Vesicular glutamate transporter 2 (VGlut2), as a part of the glutamatergic systems, seems to play a specific role. Antibodies against the VGlut2 transporter could cause synaptic dysfunction, and for the first time in the context of cognitive impairment, Hansen et al. (c) describe the diagnosis of clinically and cerebrospinal fluid (CSF)-based AD associated with VGlut2 autoantibodies. However, a pathophysiological basis as the cause of the symptoms remains speculative and cannot be proven at present, requiring further investigation in larger cohorts to be conclusive.

Due to double stigmatization (age and mental illness), insufficiently qualified personnel, and lack of care structures for behavioral and psychological disorders in dementia BPSD in acute setting, the management of BPSD is still challenging for healthcare professionals and affects the quality of care of older adults suffering from these conditions (7). The diagnosis and treatment of older patients with psychiatric illnesses require specific abilities, skills, and attitudes to adequately classify psychiatric symptoms (e.g., major depression in old age vs. late-onset bipolar disorders), manage behavioral disturbances (e.g., due to neurocognitive disorders), identify overlapping symptoms to reach the appropriate diagnosis and treatment, manage the complex clinical situations, involve multimorbidity and polypharmacy, as well as master communication and specific tasks and conflicts in the geriatric population. Moreover, some late-onset disorders are unrecognized and very often are attributed to the neurodegenerative process increasing the burden of diagnostic flow without a clear benefit for patients and their caregivers. This prejudice could delay diagnosis, exposing the patient to a risk of being labeled as suffering from another disorder or leading to chronic symptoms and lack of subsequent therapeutic success. Porpiglia et al. report the case of a patient affected by limbic encephalitis (also known as antibody-mediated encephalitis) with neuropsychiatric features at the onset. In this case report, psychiatric-onset LE clouded the clinician's judgment, leading to a misdiagnosis, delayed treatment, and poor prognosis. This attitude to attributing neuropsychiatric symptoms to aging-related neurodegenerative diseases causes patients to be diagnosed as being affected by dementia with all the negative consequences for patients and society that this entails.

## Perspective

In conclusion, this Research Topic provided significant clinical cases in the field of aging psychiatry. Further research is needed in aging psychiatry because older adults are more likely to experience mental health problems, and they may face unique challenges in accessing and receiving appropriate treatment. There is a need for a better understanding of the complex interplay between mental health and aging, the psychological substrate of late-onset behavioral disorders, as well as the specific mechanisms underlying these relationships. Fighting agism and stigma toward mental health in old age is a priority. Despite limitations in drawing clear conclusions from the series of case reports, this Research Topic expands the spectrum of mechanisms underlying neurodegenerative disorders for which there is yet no available effective treatment on a large scale. Moreover it shed light on the importance of carefully analyzing each case where neuropsychiatric manifestations are the prevailing symptoms. This will prevent misdiagnosis and deleterious results and increase knowledge in this very fascinating and demanding field. Even if more resources are available for detecting, monitoring, and treating the most common pathologies in the elderly, more efforts are needed in research and clinical practice to make aging psychiatry more acknowledged. This Research Topic of articles is moving in this direction.

## Author contributions

VF: Writing—original draft.
